# Age-dependent changes in mitochondrial morphology and volume are not predictors of lifespan

**DOI:** 10.18632/aging.100639

**Published:** 2014-02-16

**Authors:** Saroj G. Regmi, Stéphane G. Rolland, Barbara Conradt

**Affiliations:** ^1^ Center for Integrated Protein Science Munich - CIPSM, Department Biology II, Ludwig-Maximilians-University Munich, 82152 Planegg-Martinsried, Germany; ^2^ Geisel School of Medicine at Dartmouth, Department of Genetics, Hanover, NH 03755, USA

**Keywords:** C. elegans, mitochondria, aging, bio-marker, lifespan, muscle

## Abstract

Mitochondrial dysfunction is a hallmark of skeletal muscle degeneration during aging. One mechanism through which mitochondrial dysfunction can be caused is through changes in mitochondrial morphology. To determine the role of mitochondrial morphology changes in age-dependent mitochondrial dysfunction, we studied mitochondrial morphology in body wall muscles of the nematode *C. elegans*. We found that in this tissue, animals display a tubular mitochondrial network, which fragments with increasing age. This fragmentation is accompanied by a decrease in mitochondrial volume. Mitochondrial fragmentation and volume loss occur faster under conditions that shorten lifespan and occur slower under conditions that increase lifespan. However, neither mitochondrial morphology nor mitochondrial volume of five- and seven-day old wild-type animals can be used to predict individual lifespan. Our results indicate that while mitochondria in body wall muscles undergo age-dependent fragmentation and a loss in volume, these changes are not the cause of aging but rather a consequence of the aging process.

## INTRODUCTION

The age-associated degeneration of skeletal muscle function termed ‘sarcopenia’ leads to debilitating conditions among the elderly [1]. Low skeletal muscle mass or strength is believed to be the most frequent cause of disability and a major risk factor of health-related conditions [2, 3]. Various model organisms have been used to understand this age-related muscle decline [4-7]. Studies in *Caenorhabditis elegans*, for instance, have revealed that sarcopenia is also a feature of aging in nematodes [8]. Due to its short lifespan and its amenability to genetic manipulation, *C. elegans* is a suitable model to address diverse aspects of sarcopenia [8].

Studies in *C. elegans* have revealed that lifespan is influenced by gene activity, the environment, certain chemicals as well as stochastic factors [9, 10]. For example, lifespan in *C. elegans* shows a linear inverse-correlation to temperature of growth (for temperatures between 16°C to 25.5°C) [11]. Furthermore, various genetic pathways that affect lifespan have been identified. One important pathway implicated in lifespan extension is the insulin/IGF-1 signal transduction pathway [12]. The activation of the *C. elegans* IGF-1 receptor tyrosine kinase DAF-2 (DAF, dauer larva formation abnormal) induces the activation of the PI3 kinase AGE-1 (AGE, ageing alteration), which leads to the phosphorylation of the FOXO transcription factor DAF-16. When phosphorylated, DAF-16 is retained in the cytoplasm and therefore unable to modulate gene expression [9]. In response to stress, DAF-16 is dephosphorylated, and, consequently, re-localizes into the nucleus. In the nucleus, DAF-16 activates the transcription of different types of survival genes, such as genes involved in oxidative stress response, heat shock response, innate immunity, metabolism, and xenobiotic response [9]. Hence, loss-of-function mutations in the genes *daf-2* or *age-1*, lead to the nuclear localization of DAF-16 and an increased lifespan, while loss-of-function mutations in *daf-16* lead to a decreased lifespan [9, 12-16]. Furthermore, mutations in various genes encoding proteins implicated in various aspects of mitochondrial function can also lead to an increased lifespan [9, 17]. These mutations extend lifespan independently of *daf-16* and - apart from affecting lifespan - also cause slow growth and slow motility as well as a decrease in brood size [9]. For example, loss-of-function mutations in the gene *clk-1* (CLK, clock abnormal), whose gene product is required for ubiquinone biosynthesis, or loss-of-function mutations in the gene *isp-1* (ISP, iron-sulfur protein), which encodes a Rieske iron-sulfur protein of the cytochrome *bc*_1_ complex (complex III), cause longevity [17, 18].

Although numerous processes have been implicated in age-associated muscle degeneration [19, 20], mitochondrial dysfunction appears to play a prominent role [1, 21]. Studies on human muscle biopsies revealed that with age, the activities of enzymes of the tricarboxylic acid cycle decrease as do those involved in oxidative phosphorylation and ATP synthesis [1]. This overall reduction in mitochondrial function could be due to a decrease in mitochondrial volume, an increase in oxidative stress, an accumulation of mutations in mitochondrial DNA (mtDNA), and/or altered mitochondrial morphology, all of which have been observed in aging muscle cells [1, 22-24].

The role of changes in mitochondrial morphology in age-dependent mitochondrial dysfunction remains poorly understood. Electron microscopic analysis of biopsies have revealed interconnected mitochondrial networks with giant mitochondria that have ultrastructural abnormalities in skeletal muscle cells of older humans [25]. Furthermore, swollen mitochondria have been observed in aged *C. elegans* [26]. In contrast, an *in vitro* study using muscle cells derived from rat suggested that mitochondria are more fragmented in aging muscle cells [27]. The mechanisms underlying these changes in morphology also remain unclear. While an increase in the expression of the gene *Drp1* (Dynamin-related protein 1), which encodes a dynamin-related GTPase that promotes mitochondrial fission, was observed in the *in vitro* study using rat muscle cells [27], another study, also using tissues derived from rat, reported that the expression of both fission-promoting and fusion-promoting factors increased in aging muscle [28]. Furthermore, how these changes in mitochondrial morphology affect muscle cell function remains elusive [29]. However, the loss of the fusion-promoting factor Mfn2 (Mitofusin-2) in skeletal muscles causes symptoms reminiscent of sarcopenia, at least in mouse [30]. This suggests that mitochondrial fusion and, more generally, mitochondrial dynamics and morphology are important for the maintenance of muscle function.

In *C. elegans*, similar to what has been observed in human muscle biopsies, a global decrease in mitochondrial activity occurs with age. Specifically, the activity of NADH:ubiquinone oxidoreductase (complex I) decreases, as does oxygen consumption. Furthermore, with age, carbonylated proteins accumulate in mitochondria [26]. Using electron microscopy, it has also been shown that mitochondria in body wall muscles exhibit age-dependent swelling [26]. To analyze mitochondrial morphology in live animals, we generated transgenic animals that express a mitochondrial matrix-targeted GFP (mitoGFP) under the control of a body wall muscle-specific promoter. We then monitored mitochondrial morphology in animals with increasing age. Our results demonstrate that in wild type, mitochondrial length and volume decrease with age, while mitochondrial circularity increases. Furthermore, we show that the rates of these changes are altered in aging mutants and in animals raised at different temperatures. Finally, we tested whether mitochondrial length, circularity and/or volume can be used as a biomarker of aging. Our data suggests that these parameters are not predictors of lifespan in *C. elegans*.

## RESULTS

### Mitochondria in body wall muscle cells exhibit age-dependent fragmentation and volume loss

**Figure 1:**
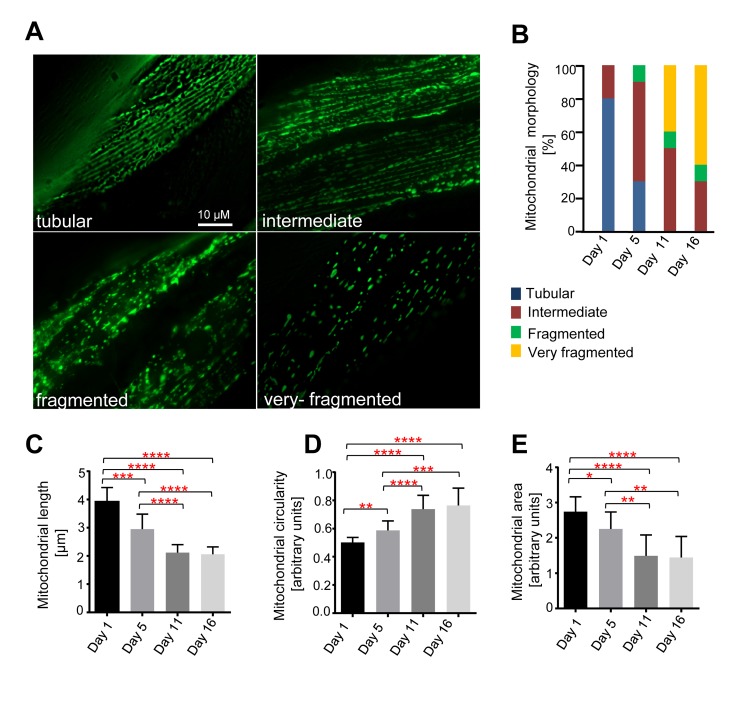
Age-dependent mitochondrial changes in *C. elegans* body wall muscle cells Transgenic animals expressing mitoGFP in body wall muscle cells (*bcIs78* [*P_myo-3_ mitoGFP*]) were analyzed at different days after the L4 larval stage (Day 1, Day5, Day 11 and Day 16). (**A**) Representative images of the different mitochondrial morphologies observed. (**B**) Qualitative analysis of mitochondrial morphology with age. Blue, Red, Green and Yellow represent percentage of animals displaying tubular, intermediate, fragmented and very fragmented mitochondrial morphology, respectively (n=10-13 images). Using the same dataset, mitochondrial length (n=10-13 cells) (**C**), circularity (n=10-13 cells) (**D**) and area (n=9-11 images) (E) were measured. Error bars indicate standard deviations. Statistical significance was tested using the Student t-test (* p<0.05, ** p<0.01, *** p<0.001 and **** p<0.0001).

To monitor mitochondrial morphology in body-wall muscle cells with age, we used a strain expressing mitochondrial matrix targeted GFP (mitoGFP) under the control of the *myo-3* promoter (P*_myo-3_mitoGFP*), which is active in body wall muscle cells [31]. Although there was some variability between individual body-wall muscle cells and between individual animals, we detected age-dependent changes in mitochondrial morphology (Figure 1). Based on the different morphologies observed, we performed a double-blind qualitative assessment of mitochondrial morphology (Figure 1A, B). Consistent with previous analyses [32-34], we observed mainly tubular or intermediate mitochondrial morphology in animals 24 hs after the L4 larval stage (referred to as ‘Day 1’). At Day 5, we observed predominantly intermediate mitochondrial morphology. At Day 11, apart from tubular, intermediate and fragmented mitochondrial morphology, we also observed very fragmented mitochondrial morphology. Finally, at Day 16, we observed predominantly very fragmented mitochondrial morphology. Therefore, with age, progressive fragmentation of the mitochondrial network can be observed in body wall muscle cells of *C. elegans*.

We also quantified mitochondrial length, circularity and area (as a measure of mitochondrial volume). We observed a decrease in mitochondrial length, an increase in mitochondrial circularity, and a decrease in mitochondrial area with age (Figures 1A, C-E). We observed a significant decrease in mitochondrial length from D1-5 (Day 1 – Day 5), D1-11, and D 1-16. A comparison of mitochondrial length at Day 5 and Day 11 as well as Day 5 and Day 16 also revealed significant decreases (Figure 1C). An analysis of mitochondrial circularity revealed a significant increase from D1-5, D1-11, D1-16, D5-11 and D5-16 (Figure 1D). Finally, an analysis of mitochondrial area revealed a significant decrease from D1-5, D1-11, D1-16, D5-11, D5-16 (Figure 1E). To ascertain that these phenomena were independent of the mitochondrial reporter (P*_myo-3_mitoGFP*) and the reagent used to immobilize animals for imaging (levamisole), we confirmed these age-dependent changes in mitochondrial morphology and volume using a different mitochondrial reporter and in the absence of levamisole (data not shown). Altogether, these data demonstrate that mitochondria fragment with age as revealed by a decrease in mitochondrial length, an increase in mitochondrial circularity and an overall increasingly ‘fragmented’ morphology. Furthermore, they demonstrate that mitochondria lose volume with age as revealed by a decrease in mitochondrial area in the imaged planes.

### Age-dependent mitochondrial changes occur faster in animals raised at 25°C and slower in animals raised at 15°C

**Figure 2:**
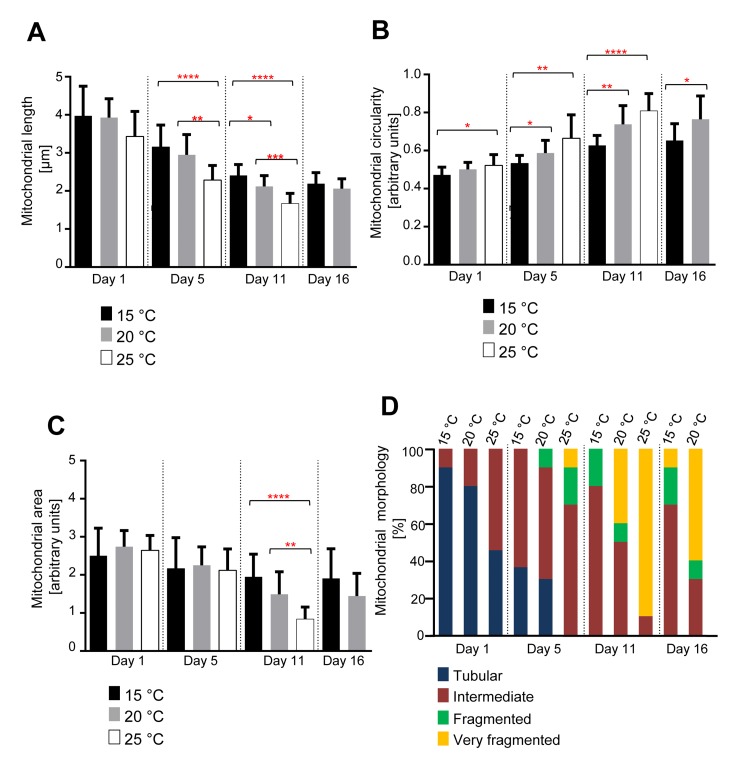
Age-dependent mitochondrial changes occur faster at 25 °C and slower at 15 °C (**A**) Mitochondrial length (n=9-15 cells), (**B**) circularity (n=9-15 cells), and (**C**) area (n=9-10 images) of animals expressing mitoGFP in body wall muscle cells (*bcIs78* [*P_myo-3_ mitoGFP*]) raised at 15°C, 20°C and 25°C. Error bars indicate standard deviations. Statistical significance was tested using the Student t-test (* p<0.05, ** p<0.01, *** p<0.001 and **** p<0.0001). (**D**) Qualitative analysis of mitochondrial morphology with age. Blue, Red, Green and Yellow represent percentage of animals displaying tubular, intermediate, fragmented and very fragmented mitochondrial morphology, respectively (n=10-11 images).

Next, we asked if there is a correlation between the changes in mitochondrial morphology and aging. *C. elegans* exhibit a shorter lifespan at 25°C in comparison to 15°C [11]. We therefore repeated the analyses with animals raised at 15°C, 20°C or 25°C. We observed that for all temperatures tested, mitochondrial length and mitochondrial area decreased with age, while mitochondrial circularity increased (Figure 2A-C). However, these changes occurred faster in animals raised at 25°C compared to animals grown at either 15°C or 20°C. While the three groups of animals displayed a similar mitochondrial length at Day 1, mitochondrial length at Day 5 was significantly shorter in animals raised at 25°C compared to animals raised at 15°C or 20°C (Figure 2A). At Day 11, mitochondrial length was significantly shorter in animals raised at 25°C compared to animals raised at 20°C and in animals raised at 20°C compared to animals raised at 15°C. At Day 16, no significant difference in mitochondrial length was detected between animals raised at 20°C and animals raised at 15°C. Mitochondrial length in animals grown at 25°C could not be analyzed because most of the animals had died prior to Day 16.

We observed a similar trend in case of mitochondrial circularity (Figure 2B). At Day 5, mitochondrial circularity was significantly different between animals raised at 15°C and animals raised at 25°C. The difference was also significant between animals raised at 15°C and animals raised at 20°C and between animals raised at 15°C and animals raised at 25°C. At Day 11, this trend continued with significant differences between animals raised at 15°C and animals raised at 20°C as well as animals raised at 15°C and animals raised at 25°C. At Day 16, a significant difference was still detected between animals raised at 15°C and animals raised at 20°C.

In the case of mitochondrial area, we only observed a significant difference at Day 11 between animals grown at 15°C compared to 25°C and animals grown at 20°C compared to 25°C (Figure 2C). The qualitative assessment confirmed the shift of mitochondrial morphology from a tubular state to a fragmented state with age at all temperatures of growth. These changes in mitochondrial morphology occur faster in animals raised at 25°C compared to animals raised at 20°C and faster in animals raised at 20°C than in animals raised at 15°C (Figure 2D). In summary, these data indicate that the rates of mitochondrial fragmentation and volume loss are inversely proportional to the temperature at which the animal is raised and therefore proportional to the rate at which the animal is aging.

### Age-dependent mitochondrial changes occur slower in long-lived *age-1(hx546)* animals

**Figure 3:**
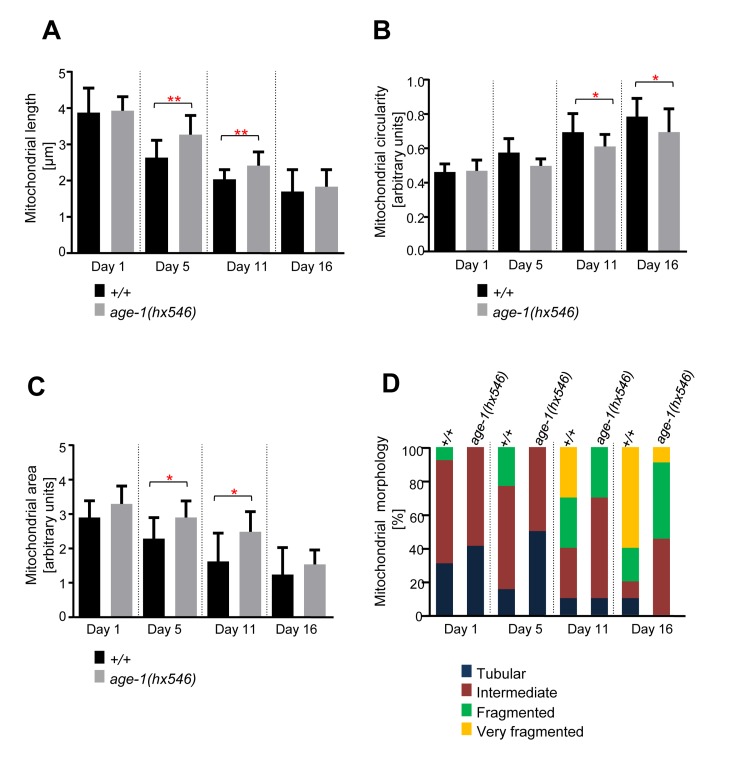
Age-dependent mitochondrial changes occur slower in *age-1(hx546)* animals Comparison of (**A**) mitochondrial length (n=12-17 cells), (**B**) circularity (n=12-17 cells), and (**C**) area (n=9-13 images) in wild-type animals expressing mitoGFP in body wall muscle cells (*bcIs78* [*P_myo-3_ mitoGFP*]) and in isogenic *age-1(hx546)* animals (*age-1(hx546); bcIs78*). Error bars indicate standard deviations. Statistical significance was tested using the Student t-test (* p<0.05, ** p<0.01, *** p<0.001 and **** p<0.0001). (**D**) Qualitative analysis of mitochondrial morphology with age. Blue, Red, Green and Yellow represent percentage of animals displaying tubular, intermediate, fragmented and very fragmented mitochondrial morphology, respectively (n=10-17 images).

To determine the effects of mutations in genes implicated in the aging process on the mitochondrial morphology observed, we repeated the analyses with animals of different genetic backgrounds. We first analyzed animals carrying a loss-of-function mutation in the gene *age-1* (*age-1(hx546)*). *age-1* encodes the *C. elegans* ortholog of the phosphoinositide 3-kinase (PI3K) and the mutation *hx546* causes an extension of lifespan by ~40% [14, 35]. We confirmed that *age-1(hx546)* animals live longer than wild-type animals (Figure S1B). We observed that mitochondrial length and area decreased with age, while mitochondrial circularity increased with age in both wild type and *age-1(hx546)* animals (Figure 3). However, these changes occurred faster in wild type than in *age-1(hx546)* animals. The difference in mitochondrial length between the two strains was significant at Day 5 and at Day 11 (Figure 3A). In case of mitochondrial circularity, the difference between the two strains was significant at Day 11 and at Day 16 (Figure 3B). The analysis of mitochondrial area revealed a similar trend as for mitochondrial length with significant differences between the two strains at Day 5 and at Day 11 (Figure 3C). The qualitative assessment of mitochondrial morphology confirmed the differences observed between wild type and *age-1*(*hx546)*. Most significantly, in *age-1*(*hx546)* animals, fragmented mitochondrial morphology was only observed at Day 11 whereas it was already observed at Day 1 in wild type (Figure 3D). In conclusion, age-dependent mitochondrial fragmentation and volume loss are significantly delayed in long-lived *age-1*(*hx546)* animals.

### Age-dependent mitochondrial changes occur faster in short-lived *daf-16(mu86)* animals

**Figure 4:**
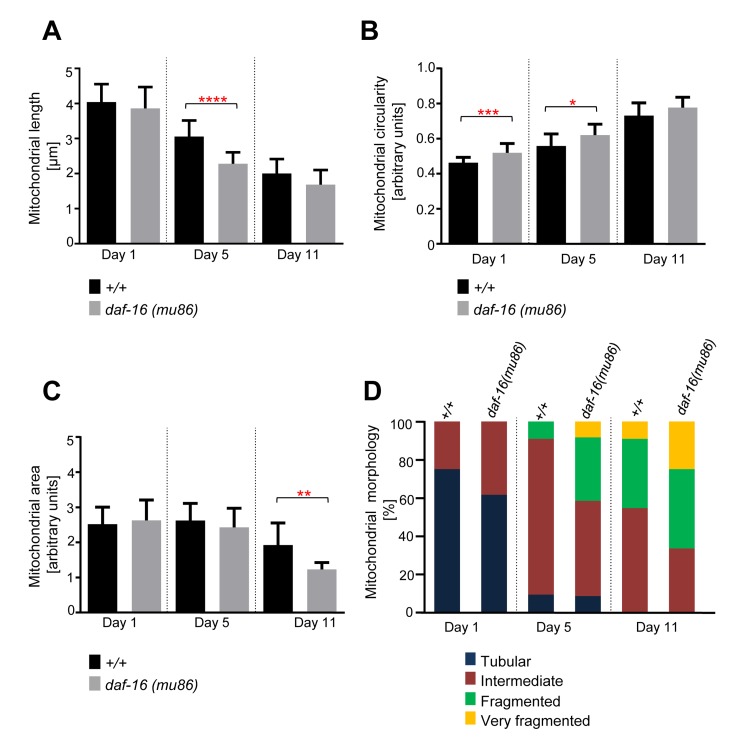
Age-dependent mitochondrial changes occur faster in *daf-16(mu86)* animals Comparison of (**A**) mitochondrial length (n=12-20 cells), (**B**) circularity (n=12-20 cells), and (**C**) area (n=11-13 images) in wild-type animals expressing mitoGFP in body wall muscle cells (*bcIs78* [*P_myo-3_ mitoGFP*]) and in isogenic *daf-16(mu86)* animals (*daf-16(mu86); bcIs78*). Error bars indicate standard deviations. Statistical significance was tested using the Student t-test (* p<0.05, ** p<0.01, *** p<0.001 and **** p<0.0001). (**D**) Qualitative analysis of mitochondrial morphology with age. Blue, Red, Green and Yellow represent percentage of animals displaying tubular, intermediate, fragmented and very fragmented mitochondrial morphology, respectively (n=11-13 images).

*daf-16* encodes a Fork-head transcription factor and a loss-of-function mutation in *daf-16* causes animals to live ~20% shorter [12, 13]. We confirmed that *daf-16(mu86)* animals live shorter than wild-type animals (Figure S1C). We observed that mitochondrial length and area decreased with age, while mitochondrial circularity increased with age in both strains (Figure 4). However, these changes occurred faster in *daf-16(mu86)* animals than in wild-type animals. For example, a significant difference in mitochondrial length was observed between wild type and *daf-16(mu86)* at Day 5 (Figure 4A). Due to the short lifespan of *daf-16(mu86)* animals, an analysis at Day 16 was not possible. An analysis of mitochondrial circularity showed a significant difference between the two strains at Day 1 and at Day 5 (Figure 4B). In addition, mitochondrial area was significantly different between the strains at Day 11 (Figure 4C). These observations were confirmed through the qualitative assessment of mitochondrial morphology. Most significantly, in *daf-16(mu86)* animals, very fragmented mitochondrial morphology was already observed at Day 5, whereas in wild type, it was only observed at Day 11 (Figure 4D). Hence, age-dependent mitochondrial fragmentation and volume loss are accelerated in short-lived *daf-16(mu86)* animals.

### Age-dependent mitochondrial changes are delayed in long-lived *clk-1(e2519)* animals

**Figure 5:**
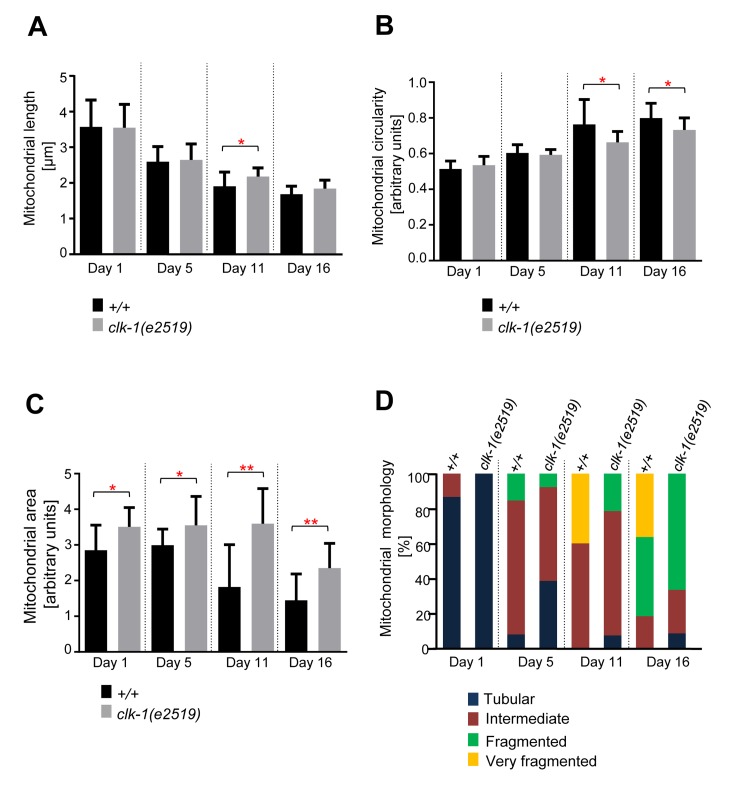
Age-dependent mitochondrial changes occur slower in *clk-1(e2519)* animals Comparison of (**A**) mitochondrial length (n=14-21 cells), (**B**) circularity (n=14-21 cells), and (**C**) area (n=9-13 images) in wild-type animals expressing mitoGFP in body wall muscle cells (*bcIs78* [*P_myo-3_ mitoGFP*]) and in isogenic *clk-1(e2519)* animals (*clk-1(e2519); bcIs78*). Error bars indicate standard deviations. Statistical significance was tested using the Student t-test (* p<0.05, ** p<0.01, *** p<0.001 and **** p<0.0001). (**D**) Qualitative analysis of mitochondrial morphology with age. Blue, Red, Green and Yellow represent percentage of animals displaying tubular, intermediate, fragmented and very fragmented mitochondrial morphology, respectively (n=10-15 images).

To test whether the age-dependent changes in mitochon-drial morphology and volume observed can solely be attributed to the IGF/insulin signaling pathway, we analyzed mitochondrial morphology in *clk-1(e2519)* mutant animals. *clk-1* encodes a hydroxylase involved in the biosynthesis of the redox-active lipid ubiquinone (co-enzyme Q) and the *clk-1* loss-of-function mutation *e2591* causes the animals to live ~25% longer than wild-type [17, 36-38]. We confirmed that *clk-1(e2591)* animals live longer than wild-type animals (Figure S1C). Similarly to what we observed with long-lived *age-1(hx546)* animals, changes in mitochondrial morphology and volume were delayed in*clk-1(e2519)* animals compared to wild-type animals (Figure 5). The difference in mitochondrial length was significant at Day 11 (Figure 5A). In case of mitochondrial circularity, the difference between the two strains was significant at Day 11 and at Day 16 (Fig. 5B).

Mitochondrial area displayed the most robust differences as they were significant between the two strains at all days analyzed (Figure 5C). These observations were confirmed through the qualitative assessment of mitochondrial morphology. Very fragmented mitochondrial morphology was observed at Day 11 and Day 16 in wild-type animals. In contrast, very fragmented mitochondrial morphology was not observed in *clk-1(e2519)* animals throughout the course of the experiment (Day 1 through Day 16) (Figure 5D). In conclusion, age-dependent mitochondrial fragmen-tation and volume loss are affected not only by mutations in the IGF/insulin pathway but also by mutations in genes that affect mitochondrial function.

### The extent of mitochondrial fragmentation and volume loss in Day 5- and Day 7-old animals cannot be used to predict lifespan

**Figure 6:**
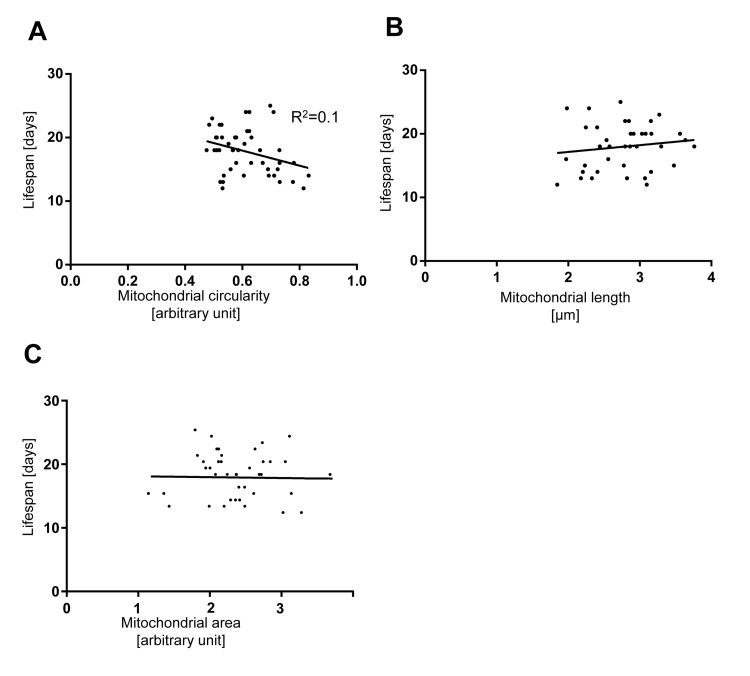
The extent of mitochondrial fragmentation at day 5 and 7 is not a bio-marker of aging Lifespan plotted against (**A**) mitochondrial circularity, (**B**) mitochondrial length, and (**C**) mitochondrial area in control *bcIs78* animals at day 5 and 7. Each dot represents a single worm.

Since we observed that mitochondria fragment and lose volume in body wall muscles with age, we tested whether the extent of mitochondrial fragmentation and volume loss in this tissue early in the life of an animal can be used to predict its lifespan. We quantified mitochondrial circularity, length and area in individual animals at Day 5 or Day 7, recovered them after imaging and determined their respective lifespan. While we observed that animals for which mitochondrial circularity was lower (i.e. less fragmented mitochondria) at Day 5 or Day 7 tend to have a longer lifespan, the overall correlation is low (R^2^ coefficient=0.1) (Figure 6A). In the case of mitochondrial length or mitochondrial area and lifespan, no correlation was detected (Figure 6B and Figure C). In conclusion, mitochondrial circularity, mitochondrial length and mitochondrial area at Day 5 and Day 7 of *C. elegans'* life cannot be used to predict individual lifespan.

## DISCUSSION

The mitochondrial ultrastructure in *C. elegans* body wall muscle cells has previously been shown to change during aging, with mitochondria becoming progressively more swollen [26]. We present new evidences that the overall morphology of the mitochondrial network changes with age. Similar to what has been observed for mitochondria in rat muscle cells [27], mitochondria in *C. elegans* muscle cells fragment with age. Furthermore, using mitochondrial area as a measure of mitochondrial volume, we found that the volume of mitochondria in muscle cells is significantly reduced in old animals when compared to young animals. Such a decrease in mitochondrial volume is expected to lead to reduced mitochondrial activity, as evidenced by the finding that increasing mitochondrial volume can lead to increased mitochondrial activity [39]. The level of connectivity of the mitochondrial network can also affect the capacity of mitochondria to produce energy [40]. For instance, the elongation of the mitochondrial network has been associated with increased energy demand, while mitochondrial fragmentation has been associated with decreased energy demand and higher energy supply [40]. Hence, an increase in mitochondrial fragmentation and a decrease in mitochondrial volume with age are likely to be causal in the age-dependent decrease in mitochondrial activity that has been observed in *C. elegans* as well as other organisms [22-24, 26].

Although a decrease in mitochondrial activity with age could explain the progressive loss in muscle function leading to sarcopenia, it has not been previously determined whether this will have a direct effect on lifespan. To address this question, we analyzed the kinetics of changes in mitochondrial morphology in different aging mutants. Interestingly, we discovered that mitochondrial fragmentation and the decrease in mitochondrial volume are accelerated in short-lived mutants and delayed in long-lived mutants. The extent of these changes therefore seems to correlate with the biological age of the animal. However, we found that the values of these parameters in a young adult animal cannot be used to reliably predict its lifespan. Therefore, our results suggest that mitochondrial morphology and volume cannot be used as bio-markers of aging. Although mitochondrial fragmentation likely causes an age-dependent decrease in mitochondrial activity, this decrease is unlikely to be the primary cause of aging as evidenced by the observation that long-lived *clk-1* animals, at least in hypodermal cells, display a faster decrease in mitochondrial membrane potential with age than compared to wild type animals [41].

Mitochondrial morphology is the result of a balance between mitochondrial fusion and fission. In *C. elegans*, mitochondrial fission is controlled by the dynamin-related GTPase DRP-1 [32]. Although the role of this protein in aging has been investigated, the results are not entirely clearcut [42, 43]. Whereas animals homozygous for the *drp-1* loss-of-function mutation *tm1108* have a normal lifespan, the inactivation of *drp-1* by RNAi has been shown to decrease lifespan [42, 43]. Despite this discrepancy, both results indicate that blocking mitochondrial fission does not extend lifespan. This supports the notion that age-dependent mitochondrial fragmentation is not a primary determinant of aging, even though it likely participates in the manifestation of some age-related symptoms such as sarcopenia. On the other hand, whether increasing mitochondrial fusion can increase lifespan and/or delay sarcopenia, remains to be determined.

Why mitochondria fragment with age remains unclear. It could be attributed to the increased oxidative stress observed in aging animals [26], as oxidative stress has been shown to induce mitochondrial fragmentation, at least in mammalian tissue culture cells [44]. This could also be an indirect effect caused by hyperactivity of mitochondria in aging leading to oxidative stress [45, 46]. Furthermore, the age-dependent mitochondrial morphology changes observed could also be a consequence of the age-dependent disorganization of the muscle structure. Interestingly, similarly to what we observed for mitochondrial fragmentation, the increased disorganization of muscle sarcomeres with age is delayed in *age-1(hx546)* animals [8]. Additionally, while mitochondrial fragmentation and, consequently, the reduction in mitochondrial activity could be an explanation for the progressive locomotory impairment observed in old animals, it is also feasible that age-dependent reduction in locomotion triggers mitochondrial fragmentation. This notion is supported by experiments performed with rats, which indicate that a reduction in physical exercise can lead to the fragmentation of the mitochondrial network in muscle cells [27]. To test whether increased mitochondrial fragmentation with age is due to decreased locomotion in aging worms, one could stimulate “exercise” in aging *C. elegans* animals, for example by raising them in liquid medium, in which *C. elegans* animals are thought to be more active. Conversely, one could test whether reducing *C. elegans* locomotion (by using mutants or drugs) will accelerate the fragmentation of the mitochondrial network and possibly decrease lifespan.

Finally, the mechanism or mechanisms through which mitochondrial volume decreases with age remain to be determined; however, we speculate that a decrease in mitochondrial volume could be caused by a reduction in mitochondrial biogenesis [1]. Alternatively, since we measured mitochondrial area in a defined region of the muscle cell (region of 300 × 300 pixels) and since muscle cell size continues to increase with age, we cannot exclude that the observed decrease in mitochondrial volume is - at least in part - due to an increase in muscle cell size.

The increased understanding of the control of longevity has generated considerable interest in the identification of biomarkers of aging. Studies using various model organisms and human tissue samples have led to the identification of a few biomarkers of aging that can predict lifespan with some success. For example, the state of DNA methylation in various tissues in humans and the length of telomeres in zebra-finches can be used as biomarkers of aging [47, 48]. In *C. elegans*, it has been shown that the major source of lifespan variability is different pathogenicity from individual to individual [49]. Furthermore, a recent study revealed that in *C. elegans,* the production of cross progeny production through Day 4 is an early-stage biomarker of longevity [50]. Interestingly, microRNA reporters (*mir-71*::GFP, *mir-246*::GFP and *mir-239*::GFP) can also be used to predict lifespan in *C. elegans* [51]. In our study we tested whether mitochondrial fragmentation (as measured by length, circularity, morphology and mitochondrial volume loss) can be used as a biomarker of aging. While we observed that mitochondria undergo fragmentation and loss of volume with age, our results show that these mitochondrial changes cannot be used as a predictor of lifespan.

In summary, we show that mitochondrial fragmentation and a decrease in mitochondrial area (as a read out of decrease in mitochondrial volume)correlate with age and are likely to participate in the degeneration of body wall muscles. We also present evidences that these changes are unlikely to be the primary cause of aging. Future studies are warranted to understand how these mitochondrial changes are induced during aging with the aim of increasing our understanding of sarcopenia and other age-related conditions.

## METHODS

### General *C. elegans* methods and strains

*C. elegans* strains were maintained and cultured as described previously [52]. Mutations used in this study have been previously described and are the following: LG I, *daf-16(mu86)*[13] LG II, *age-1(hx546)*[35], LG III, *clk-1(e2519)* [17]. Bristol N2 was used as the wild-type strain. PCR amplification combined with DNA sequencing was performed to validate genotypes when necessary. *bcIs78* is an integration of the P*_myo-3_mitoGFP* reporter allowing visualization of mitochondria in body wall muscle cells [31]. To ensure that the aging studies were performed with isogenic strains, *bcIs78* males were crossed with hermaphrodites of the different mutant strains used in the study. F2 animals homozygous for both, *bcIs78* and the respective mutation as well as F2 animals homozygous for *bcIs78* only (wild-type control) were selected for studies with aging mutants.

### Analysis of mitochondrial length and circularity in body wall muscle cells

Mitochondrial length and circularity in body wall muscle cells were measured as described previously [33]. Mitochondrial circularity is a measure of “roundness” of mitochondria with 0 referring to a straight line and 1 as a perfect circle. Animals were grown at 20 degrees. For the temperature shift experiment, animals were raised at 20 degrees until they reached the L4 larval stage and then shifted to the appropriate temperature (as indicated in the text). Adults were allowed to lay eggs for 3-5 hours and their progeny at the L4 stage were transferred to a new plate. At least 10 animals were imaged at specific days (Day 1, Day 5, Day 11 and Day 16) by fluorescence microscopy, in order to be able to analyze mitochondrial morphology in at least 10 muscle cells for each time point. Mitochondrial length and circularity in muscle cells was measured using Metamorph software on deconvolved images. The different datasets follow a normal distribution. The resulting data were visualized using GraphPad Prism Software and analyzed by Student's *t-*test.

### Analysis of mitochondrial area in body wall muscle cells

The same image datasets were used for analysis of mitochondrial area in body wall muscle cells. A 300 × 300 pixels region was selected from each image and mitochondrial area was determined by measuring the fluorescent area of the pixel region (pixel area) using Metamorph software. The data analysis was done double blind and average of both datasets was used for comparison. At least 9 images were analyzed for each time point per genotype.

### Qualitative assessment of mitochondrial morphology in body wall muscle cells

Qualitative assessment of mitochondrial morphology in body wall muscle cells was performed on the same image datasets using the whole image. Morphological categories were defined as follows: (1) images containing a majority of long interconnected mitochondrial networks were classified as *tubular*; (2) images containing a combination of interconnected mitochondrial networks along with some smaller fragmented mitochondria were classified as *intermediate*; (3) images with a majority of short mitochondria were classified as *fragmented*; and finally, (4) images with sparse small round mitochondria were classified as *very fragmented*, as shown in Figure 1A. The data analysis was performed in a blind manner.

### Lifespan analysis

Animals were grown at 20°C unless otherwise stated on NGM plates without using FUDR (5-fluorodeoxyuridine) and picked to fresh NGM plates after the L4 stage. The animals were subsequently picked to fresh NGM plates every two days until parched. Animals were scored by gentle prodding with a platinum wire. Injured, missing or bagged animals were censored. Graphpad Prism Software was used for Kaplan-Meier survival curve and log-rank test.

### Lifespan prediction experiment

*bcIs78* animals were grown at 20°C on NGM plates without using FUDR (5-fluorodeoxyuridine ) and imaged at Day 5 and Day 7. The imaged animals were recovered from the agar pad and transferred onto individual NGM plates. Animals were scored by gentle prodding with a platinum wire. Animals that were injured during the transfer after imaging were not used for the analysis. The resulting data were visualized and the goodness of fit of linear regression was analyzed using Graphpad Prism Software.

### Image acquisition and processing

Imaging was performed at 20°C using a microscope equipped with a 100× 1.3 NA oil lens (Axioskop 2; Carl Zeiss, Inc.) and a charge-coupled device camera (1300; Micromax). MetaMorph software (version 7.1; MDS Analytical Technologies) was used to acquire fluorescent z stacks of individual animals (0.5 μm/slice). The acquisition time was 100 ms. Worms were immobilized during imaging using 25mM levamisole. Deconvolution was performed blind on the fluorescent z stack (10 iterations) using AutoDeblur/AutoVisualize software (version 1.4.1; Media Cybernetics). Images shown in the figures correspond to single plane images.

## SUPPLEMENTAL FIGURE


