# Supplementary material for: Age-dependent changes in mitochondrial morphology and volume are not predictors of lifespan
Source: Aging (Albany NY). 2014 Feb 16;6(2):118–30. doi: 10.18632/aging.100639 (PMC3969280; doi:10.18632/aging.100639)
Supplement: Supplementary file 1 [file aging-06-118-s001.pdf]

49. Sanchez-Blanco A and Kim SK. Variable pathogenicity determines individual lifespan in *Caenorhabditis elegans*. PLoS Genet. 2011; 7:e1002047.
50. Pickett CL, Dietrich N, Chen J, Xiong C and Kornfeld K. Mated Progeny Production Is a Biomarker of Aging in *Caenorhabditis elegans*. G3 (Bethesda). 2013; 3:2219-2232.
51. Pincus Z, Smith-Vikos T and Slack FJ. MicroRNA predictors of longevity in *Caenorhabditis elegans*. PLoS Genet. 2011; 7:e1002306.
52. Brenner S. The genetics of *Caenorhabditis elegans*. Genetics. 1974; 77:71-94.

SUPPLEMENTAL FIGURE

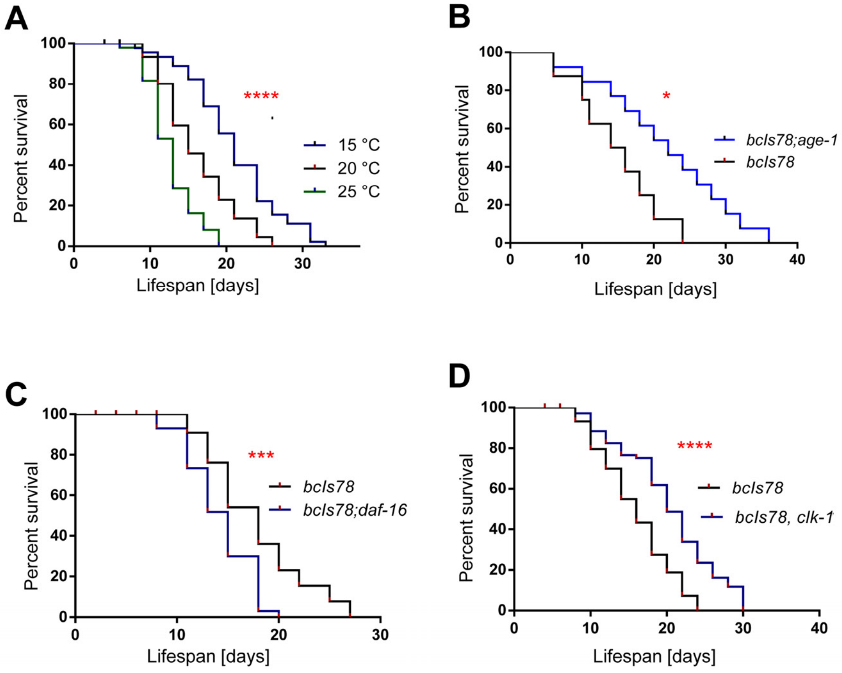

Figure S1. Lifespan data of animals analyzed in Figures 2-5. Kaplan-Meier Survival plots for +/+ (*bcIs78)* animals grown at (**A**) 15°C, 20°C and 25°C, (**B**) isogenic *bcIs78* and *bcIs78; age-1(hx546)* animals, (**C**) isogenic *bcIs78* and *bcIs78; daf-16(mu86)* animals and (**D**) isogenic *bcIs78* and *bcIs78; clk-1(e2519)* animals.
